# The Use of Technology to Provide Mental Health Services to Youth Experiencing Homelessness: Scoping Review

**DOI:** 10.2196/41939

**Published:** 2023-01-16

**Authors:** Shalini Lal, Sarah Elias, Vida Sieu, Rossana Peredo

**Affiliations:** 1 School of Rehabilitation Faculty of Medicine University of Montréal Montréal, QC Canada; 2 Youth Mental Health and Technology Lab Health Innovation and Evaluation Hub University of Montréal Hospital Research Centre Montréal, QC Canada; 3 Douglas Mental Health University Institute Montréal, QC Canada

**Keywords:** digital equity, homelessness, telemedicine, telehealth, cellular phone, internet, e-mental health, digital health, mobile health, mHealth, literature review, mobile phone

## Abstract

**Background:**

There is growing interest in using information and communication technologies (ICTs) to improve access to mental health services for youth experiencing homelessness (YEH); however, limited efforts have been made to synthesize this literature.

**Objective:**

This study aimed to review the research on the use of ICTs to provide mental health services and interventions for YEH.

**Methods:**

We used a scoping review methodology following the Arksey and O’Malley framework and guidelines from the Joanna Briggs Institute Manual for Evidence Synthesis. The results are reported according to the PRISMA (Preferred Reporting Items for Systematic Reviews and Meta-Analyses) statement and the PRISMA-ScR (Preferred Reporting Items for Systematic Reviews and Meta-Analyses extension for Scoping Reviews). A systematic search was conducted from 2005 to 2021 in MEDLINE, Embase, CINAHL, PsycInfo, Cochrane, Web of Science, and Maestro and in ProQuest Thesis and Dissertations, Papyrus, Homeless Hub, and Google Scholar for gray literature. Studies were included if participants’ mean age was between 13 and 29 years, youth with mental health issues were experiencing homelessness or living in a shelter, ICTs were used as a means of intervention, and the study provided a description of the technology. The exclusion criteria were technology that did not allow for interaction (eg, television) and languages other than French or English. The data were analyzed using descriptive statistics and qualitative approaches. Two reviewers were involved in the screening and data extraction process in consultation with a third reviewer. The data were summarized in tables and by narrative synthesis.

**Results:**

From the 2153 abstracts and titles screened, 12 were included in the analysis. The most common types of ICTs used were communication technologies (eg, phone, video, and SMS text messages) and mobile apps. The intervention goals varied widely across studies; the most common goal was reducing risky behaviors, followed by addressing cognitive functioning, providing emotional support, providing vital resources, and reducing anxiety. Most studies (9/11, 82%) focused on the feasibility of interventions. Almost all studies reported high levels of acceptability (8/9, 89%) and moderate to high frequency of use (5/6, 83%). The principal challenges were related to technical problems such as the need to replace phones, issues with data services, and phone charging.

**Conclusions:**

Our results indicate the emerging role of ICTs in the delivery of mental health services to YEH and that there is a high level of acceptability based on early feasibility studies. However, our results should be interpreted cautiously, considering the limited number of studies included in the analysis and the elevated levels of dropout. There is a need to advance efficacy and effectiveness research in this area with larger and longer studies.

**International Registered Report Identifier (IRRID):**

RR2-10.1136/bmjopen-2022-061313

## Introduction

### Background

Homelessness is a rapidly growing phenomenon that is estimated to affect approximately 1.6 billion people worldwide by 2025 [1]. It is of particular concern that youth represent 20% to 30% of the homeless population in developed countries (ie, Canada and Australia) [2,3]. The factors that contribute to homelessness in this population are multifaceted, such as lack of affordable housing or social support, barriers to completing education, economic insecurity, family conflict or domestic violence, addiction, and involvement in the child welfare system [4,5].

Moreover, because of their precarious situation, youth experiencing homelessness (YEH) may not develop the skills needed for a healthy and secure transition to adulthood [6]. Indeed, YEH are at a high risk of physical and mental health problems [7-9], with potential consequences such as nutritional vulnerability, drug use, exposure to premature sexual activity, physical abuse, sexual abuse, criminal victimization, dropping out of school, boredom, and poor access to the resources needed to maintain a satisfactory standard of living [10-14]. However, despite the clear need to provide easy access to mental health services, the traditional health care system is challenged by the nomadic lifestyle of YEH, compounding the difficulties in reaching and engaging this marginalized population [15,16].

High rates of access and use of technology among YEH have been reported in the literature, even among very young populations [17], and therefore, may be a medium for health care providers to reach and engage YEH [18-20]. For instance, a recent scoping review [20] found high percentages (ranging from 46.7% to 100%) of mobile phone ownership among 16 YEH samples and reported that, on average, 77.1% (range 57%-90.7%) of the samples used social media. This indicates that YEH are receptive to using information and communication technologies (ICTs) in their daily lives and for health purposes.

Indeed, there is growing interest in the potential use of ICTs to deliver health and mental health–related services and interventions to low-income and disadvantaged populations [18-24]. Systematic reviews indicate that the most common type of ICTs for mental health explored in recent years are videoconferencing, SMS text messaging, and mobile apps [20,25,26], but it should be noted that most reviews do not focus on the YEH population.

### Research Questions

Given the emerging research on this topic and the need to synthesize this literature to inform future policy, practice, and research, we conducted a scoping review to answer the following question: What is known about the use of technology to provide mental health services and interventions to YEH aged between 13 and 29 years?

To answer this research question, we aimed to

1. describe the type of ICTs, goal, and type of service or intervention (eg, information, education, therapy, or peer-support); prescribed frequency of use; characteristics (eg, self-directed, coached, or type of professional delivering the service); and technology type (eg, phones or web-based applications).
2. describe the available evidence on technology-based mental health interventions (including acceptability, feasibility, security, and effectiveness).
3. document the quality of the available evidence.
4. identify the implications of this evidence for mental health services.

## Methods

### Overview

This study was conducted according to the scoping review framework suggested by Arksey and O’Malley [27], the methodological guidelines of Levac et al [28], and the Joanna Briggs Institute Manual for Evidence Synthesis [29]. The protocol was developed a priori, registered retrospectively on the Open Science Framework [30], and published under peer review [31]. Results are reported according to the PRISMA (Preferred Reporting Items for Systematic Reviews and Meta-Analyses) statement for reporting systematic reviews [32] and scoping reviews (PRISMA-ScR [Preferred Reporting Items for Systematic Reviews and Meta-Analyses extension for Scoping Reviews]) [33]. The completed PRISMA-ScR checklist is provided in Multimedia Appendix 1.

### Information Sources and Search Strategy

To identify all relevant studies, a literature search was conducted in the following electronic databases: MEDLINE, Embase, CINAHL, PsycInfo, Cochrane, Web of Science, and Maestro, mainly for peer-reviewed articles; and ProQuest Thesis and Dissertations, Papyrus, Homeless Hub, and Google Scholar for gray literature. No methodological restrictions were applied, and all publications in English or French, from January 1, 2005, to May 28, 2021, were included. Publication date restrictions were chosen, given that the evolution of technology limits the applicability of the literature search; thus, literature published before 2005 would not be as pertinent to the current landscape of research and practice. Abstracts and conference presentations were included only if the authors reported sufficient details to address the objectives of this review.

The search strategy was developed through consultation with an information specialist. It was adapted from our previous scoping review [20] that focused on the access and use of technology among YEH by adding the concept of mental health. Most databases were searched on May 28, 2021, except for Web of Sciences, which was searched on August 9, 2021. Textbox 1 presents the search strategy used in MEDLINE.

**Search strategy used in MEDLINE (the search strategy was adapted for each database under the supervision of an information specialist).**

(exp “Internet” OR exp “Cell Phone” OR exp “Smartphone” OR exp “Computers” OR exp “Computers Handheld” OR exp “Mobile Applications” OR exp “Telemedicine” OR exp “Social Media” OR exp “Technology” OR exp “Medical Records Systems, Computerized” OR exp “Electronic Mail” OR exp “Social Networking” OR exp “User-Computer Interface” OR exp “Web Browser” OR exp “Virtual Reality Exposure Therapy” OR exp “Virtual Reality” OR exp “Computer Simulation” OR exp “video-audio media” OR exp “Robotics” OR exp “Wearable Electronic Devices” OR exp “Biosensing Techniques” OR exp “Video Recording” OR exp “Online Systems” OR (“internet” or “cell phone*” or “cellphone*” or “mobile phone*” or “smart phone*” or “smartphone*” or “mobile app*” or “computer*” or “social media*” or “Facebook” or “text messag*” or “electronic messag*” or “electronic mail*” or “email*” or “e-mail*” or “social network*” or “technolog*” or “electronic case* management” or “web” or “website*” or “virtual reality” or “mobile device*” or “video*” or “portal*” or “chatbot*” or “robot*” or “wearable device*” or “sensor” or “sensors” or “biosensoror” or “bio-sensor” or “biosensors” or “bio-sensors” or “smartwatch*” or “smart watch*” or “ereferr*” or “e-referr*” or “chat” or “online” or “instant messag*” or “cyber*” or “avatar*” or “platform*” or “telehealth*” or “tele-health*” or “telepsychiatry” or “tele-psychiatry” or “telepsychology” or “tele-psychology” or “telemental health*” or “tele-mental health*” or “teletherapy” or “tele-therapy” or “telemedicine” or “tele-medicine” or “telerehabilitation” or “tele-rehabilitation” or “emental health*” or “e-mental health*” or “ehealth*” or “e-health*” or “mhealth*” or “m-health*”). ab,kf,kw,ti) AND (exp “Homeless Persons” OR (“homeless*” or “street* youth*” or “street* adolescen*” or “street* teen*” or “runaway youth*” or “runaway adolescen*” or “runaway teen*” or “street* living youth*” or “street* living adolescen*” or “street* living teen*”). ab,kf,kw,ti), limit to (yr=“2005 -Current” and (english or french))

### Selection of Sources of Evidence

All references generated by the electronic search were exported into Covidence systematic review software. After duplicates were removed, 2 authors (SE and VS) independently screened all titles and abstracts based on the inclusion and exclusion criteria. Next, full-text screening was conducted by the same 2 authors. All discrepancies were resolved through discussion, and a third author (SL) was included in the case of disagreement until consensus was reached. The third author (SL) reviewed all included papers in relation to the inclusion and exclusion criteria as a final validation step.

Inclusion criteria were as follows: (1) primary studies reporting on participants with a mean age between 13 and 29 years; (2) youth with any mental health issue experiencing homelessness (including living in a shelter); (3) use of ICTs as a means of intervention to address mental health treatment, mental health promotion, socioeconomic determinants pertinent to mental health, or daily activities such as maintaining housing, returning to school or work, and so on; and (4) a description of the technology used. Studies were excluded if the authors used technology that did not allow the user to interact with it (eg, CDs, projectors, or television) and literature written in languages other than French or English.

### Data Extraction and Synthesis

The data extraction form was adapted from our previous scoping review [20] using Microsoft Excel. The form was piloted by 2 authors (SE and VS) who independently extracted data from 3 studies and compared their results in consultation with a third author (SL). Revisions were made to ensure that relevant information pertinent to the objectives was included.

The following data were extracted: (1) study characteristics, including authors, publication year, country of publication, study objectives, study design, methods, sample size, sociodemographic characteristics, dropout percentages, and follow-up; (2) intervention characteristics, including type of ICTs, technology features, implementation, and length and frequency of intervention; and (3) specific outcomes of the intervention according to the feasibility study framework by Bowen et al [34], which includes acceptability, degree of success of execution, and failure of execution. The data extraction process was conducted separately by 2 authors (SE and VS), and each of these authors validated the other’s data extraction and resolved any discrepancies. The completed data extraction form was validated by another author (RP). Publication reporting of data from the same study sample was considered a set and assessed as one study.

Data were summarized in tables and by a narrative synthesis organized according to themes pertaining to the objectives of the scoping review. Statistical analyses included simple weighted averages for continuous data (participant age), and frequencies and percentages were calculated for all other categorical data. Qualitative data (eg, intervention characteristics, acceptability, and implementation) were coded and categorized by RP and then validated by SL.

### Quality Appraisal

The methodological quality of the included studies was assessed to obtain a general idea regarding the quality of the publications. This was performed by an author (RP) using the critical appraisal tools provided by the Joanna Briggs Institute [35]. Meta-biases such as publication bias and selective reporting were not analyzed. The type of evidence was categorized based on the Joanna Briggs Institute Manual for Evidence Synthesis [35].

## Results

### Overview

**Figure 1:**
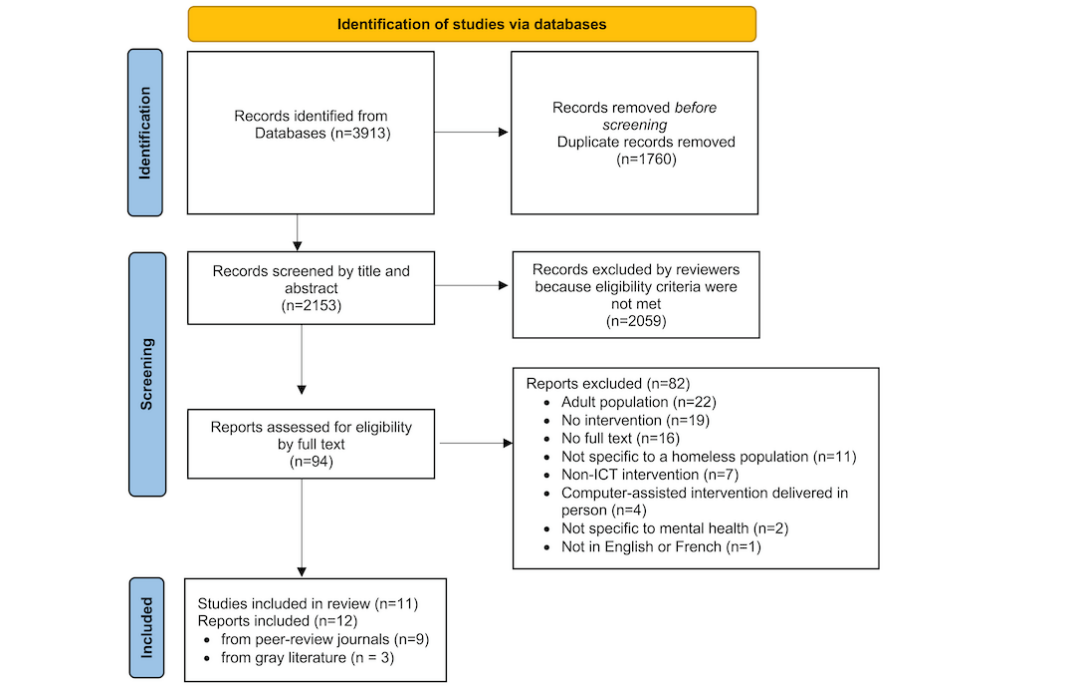
PRISMA (Preferred Reporting Items for Systematic Reviews and Meta-Analyses) flowchart for study selection. ICT: information and communication technology.

An electronic search identified 2153 unique records. After the first level of title and abstract screening, 4.37% (94/2153) of documents were retained for the second screening level that included a full-text review. Of these, 13% (12/94) of reports were selected for inclusion in the scoping review. The interrater reliability coefficient indicated moderate agreement for the first screening level (Cohen κ=0.4138) and almost perfect agreement for the second level (Cohen κ=0.877). Two reports (a poster and published abstract) identified during the screening process described the same study and population [36,37]. We decided to focus mainly on data from the poster [36] because it contained the most information. In addition, 2 other reports, Linnemayr et al [38] and Tucker et al [39], reported data concerning the same study intervention but including different sample populations. Tucker et al [39] focused on refining the intervention by examining its acceptability and feasibility, while Linnemayr et al [38] conducted a pilot study assessing the same intervention with a larger sample. We considered these to be 2 different studies only when new information was added. In total, 12 reports from 11 studies were included in the analysis. Further details about the screening process are described in the PRISMA flowchart in Figure 1. The flowchart is in accordance with the PRISMA 2020 statement for reporting systematic reviews [32].

### Overview of the Studies

All studies were conducted in North America, with the majority (10/11, 91%) conducted in the United States (Chicago, Los Angeles, Oakland, and other nonspecified cities located in the northeast and midwestern states) and one conducted in Canada (abstract; study location unspecified). Although our search strategy was extended to 2005, all reports were published between 2016 and 2021 and thus published within the last 5 years of conducting the scoping review. In terms of the types of documents reviewed, the majority (9/12, 75%) of the reports were peer-reviewed articles, and the rest were conference abstracts (2/12, 17%) and a poster (1/12, 8%). All studies were conducted in the early phase of the research (eg, intervention development, feasibility, and preliminary efficacy). Additional details of the study characteristics are provided in the latter part of the Results section.

### Sample Characteristics

Sample characteristics are presented in Table 1. Sample sizes across studies ranged from 6 to 99 participants, resulting in a total sample of 526 participants included in this scoping review. Participant ages ranged from 13 to 25 years across studies; 42.8% (225/526) identified as women, 41.1% (216/526) as men, and 2.9% (15/526) as transgender or gender fluid. Among the 3 studies that reported on sexuality [38,40,41], most participants (135/526, 25.7%) were heterosexual, 3.2% (17/526) were homosexual or bisexual, and 5.1% (27/526) reported other as sexuality. A total of 9 studies [19,36,38,40-45] reported ethnicity; 42.9% (226/526) of the participants were Black and African American, 11% (58/526) were Hispanic and Latin American, 9.3% (49/526) were White, 6.8% (36/526) were mixed, and 4.9% (26/526) were other ethnicities. Regarding mental illness conditions, 6 studies [19,40,43-46] included participants who were diagnosed with a psychiatric disorder (dysthymia, depression and bipolar disorder, anxiety, posttraumatic stress disorder, adjustment disorder, psychosis, attention-deficit hyperactivity disorder, personality disorders, or emotion regulation issues), 5 studies (6 reports) [36,38,39,42,44,45] included participants who used or abused substances such as alcohol, tobacco, or marijuana, and 2 studies [41,43] included only participants without any type of mental disorder. Table 1 also shows that 4 studies [19,36,40,42] recruited participants from homeless shelters, and the rest recruited participants from transitional housing [43,45], drop-in center [38,39,44], or various other services [40,41] for YEH.

**Table 1:** Sample demographic and study characteristics of the 11 studies included in the review^a^.

Characteristics		Values
Sample^b^ size (n), range		6-99
Age (years), range^c^		13-25
**YEH^d^ sample from studies that examined gender (n=526), n (%)**		
	Women	225 (42.8)
	Men	216 (41.1)
	Transgender	10 (1.9)
	Gender fluid	5 (1)
	Unknown or missing information	70 (13.3)
**YEH sample from studies that examined sexuality (n=526), n (%)**		
	Heterosexual	135 (25.7)
	Homosexual	9 (1.7)
	Bisexual	8 (1.5)
	Other	27 (5.1)
**YEH sample from studies that examined ethnicity (n=526), n (%)**		
	Black	226 (42.9)
	Hispanic	58 (11)
	White	49 (9.3)
	Mixed race	36 (6.8)
	Other	26 (4.9)
	Unknown or missing information	27 (5.1)
**Studies that specified mental illness or conditions^e^ (n=11), n (%)**		
	Psychiatric disorders (dysthymia, depression, bipolar disorder, anxiety, PTSD^f^, adjustment disorders, psychosis, ADHD^g^, emotion regulation, or personality disorders)	6 (54.5)
	Substance use (alcohol, tobacco, or marijuana)	5 (45.5)
	None	2 (18.2)
**Studies that specified places of YEH recruitment (n=11), n (%)**		
	Shelter	4 (36.4)
	Transitional housing	2 (18.2)
	Drop-in centers	2 (18.2)
	Various services for YEH	2 (18.2)

^a^Tucker et al [39] and Linnemayr et al [38] analyzed the same population. This table only includes data from the study by Linnemayr et al [38], because this report had the largest population.

^b^Total number of participants allocated to each intervention and those who completed baseline assessments.

^c^Age range was obtained from the age ranges provided as inclusion criteria by each study, except for the studies by Glover et al [40] and Thompson et al [42], who reported the actual sample age range. Two studies [36,47] did not report on age ranges, but their mean ages were 19.13 (SD 0.9) and 17.5 (SD 1.1) years, respectively.

^d^YEH: youth experiencing homelessness.

^e^A total of 11 studies reported on mental illness, among which 6 reported on participants with psychiatric disorders and 5 on substance use.

^f^PTSD: posttraumatic stress disorder.

^g^ADHD: attention-deficit/hyperactivity disorder.

### Study and Intervention Characteristics

The study and intervention characteristics are summarized in Table 2. All studies were in preliminary phases; the majority (9/11, 82% studies or 10 reports) aimed to assess the feasibility of the intervention [19,38-46], 3 studies explored preliminary results of efficacy [36,42,47], and 2 studies described the development of the intervention [39,41]. Three studies (4 reports) were designed as randomized controlled trials (RCTs) [38,42,44,45] but only reported feasibility or preliminary results of efficacy. The remaining studies (7/11, 64% studies) were experimental trials without randomization or were quasi-experimental studies [19,36,39-41,43,46]. In terms of the intervention’s principal aim, 4 studies (5 reports) [36,38,39,42,47] described interventions that were intended to reduce risky behaviors such as substance consumption (including tobacco, alcohol, and marijuana) and sexual risk behaviors, 3 interventions [40,45,46] focused on cognitive functioning, 3 interventions [19,40,43] aimed to provide emotional support or help with emotion regulation, 2 interventions [40,42] provided information about access to health or other vital resources for YEH, and 1 intervention [44] aimed to reduce anxiety and physiological stress.

Intervention length varied greatly across the studies from 1 week to 8 months, and 2 studies offered a single-time use intervention [41,43]. Only 2 studies [38,45] included a follow-up period of 1 and 3 months. There was also great variation in dropout percentages from 14% to 81% among the 7 studies [19,38-40,42,43,45] that provided this information. From these studies, only 3 reported the reasons for dropout [42,43,45]; the most common reasons were loss of interest, discharges, scheduling conflicts, and change of address (Table 3).

**Table 2:** Study and intervention characteristics organized by level of evidence.

	Study characteristics			Intervention characteristics		
	Design	Study aim	Technology		Features	Intervention aim
Thompson et al [42], 2020	RCT^a^	Feasibility and preliminary efficacy	Smartphone or mobile apps		Daily tracker, in-person counseling, and resources information	Reduce substance use and risky sexual behaviors
Medalia et al [45], 2017	RCT	Feasibility	Computer		Computer exercises	Training in neurocognition
Linnemayr et al [38], 2021	Pilot RCT	Feasibility	Mobile phone		SMS text messages	Quit smoking
Tucker et al [39], 2020	Quasi-experimental	Development feasibility	Mobile phone		SMS text messages	Quit smoking
Chavez et al [44], 2020	Pilot RCT	Feasibility	Oculus Go headset		Virtual reality	Reduce anxiety and physiological stress
Schueller et al [19], 2019	Quasi-experimental	Feasibility	Smartphone or mobile apps		Push-in tips, daily tracker, phone calls, and SMS text messages	Brief emotional support and coping skills
Glover et al [40], 2019	Quasi-experimental	Feasibility	Smartphone and mobile apps		Push-in tips, daily tracker, hotline, text messages, automated systems, and resources information	Mental health resources, emotional support, and brief cognitive behavioral interventions
Leonard et al [43], 2018	Quasi-experimental	Feasibility	Smartphone and mobile apps and wrist-worn sensor band		Daily tracker, push-in tips, and self-report alert	Emotion regulation (adolescent mothers)
Sheoran et al [41], 2016	Quasi-experimental	Development and feasibility	Smartphone and mobile apps		Resources information	Access to health and vital resources
Chao et al [36], 2017	Quasi-experimental^b^	Efficacy	Smartphone and mobile apps		Phone or video calls and push-in tips	Reduce alcohol and substance use
Karnik et al [46], 2017	Quasi-experimental^c^	Feasibility	Smartphone and mobile apps		Phone calls and tracker	Cognitive behavioral therapy
Archie et al [47], 2018	Quasi-experimental^c^	Preliminary evidence on efficacy	Video game		N/A^d^	Raise awareness of marijuana use

^a^RCT: randomized controlled trial.

^b^Poster.

^c^Conference abstract.

^d^N/A: not applicable.

**Table 3:** Participant engagement and retention strategies.

Intervention type, reference		Participants, n	Intervention		Dropout^a^, n (%)		Reported reasons to drop out	Financial or material support	Access to the technology	
			Duration	Follow-up						
**Smartphone**										
	Thompson et al [42], 2020	60	28 days	N/A^b^	20 (33)		16 discharged; 1 incarcerated; 1 hospitalized; 1 self-withdrew; 1 relocated	US $25 to US $50 gift cards+smartphone or US $100 gift card		Researchers provided the smartphones
	Schueller et al [19], 2019	35	1 month	N/A	15 (43)		Unclear^c^	Phone service and data		Researchers provided the smartphones
	Glover et al [40], 2019	100	6 months	N/A	81 (81)		Unclear	Smartphone+US $5 gift card		Researchers provided the smartphones
	Sheoran et al [41], 2016	6	One time	N/A	N/A		N/A	US $100 gift card		Researchers provided the smartphones. A total of 83% (5/6) participants already owned a smartphone
	Chao et al [36], 2017	24	1 month	N/A	Unclear		Unclear	Unclear		Researchers provided the smartphones
	Karnik et al [46], 2017	20	Unclear	Unclear	N/A		N/A	Unclear		Researchers provided the smartphones
	Leonard et al [43], 2018	49	Variable (6-8 months)	N/A	9 (18)		4 discharged; 4 technology problem; 1 did not use the ICT^d^	US $90 stipend		Researchers provided the smartphones
	Linnemayr et al [38], 2021^e^	77	6 weeks	3 months	11 (14)		Unclear	US $65		Participants’ phone
	Tucker et al [39], 2020^e^	28^f^	1 week	N/A	2 (20)^f^		Unclear	Unclear		Participants’ phone
**Other ICTs**										
	Medalia et al [45], 2017	91	26 weeks	1 month	69 (76)		Baseline to week 26: 25 loss of interest; 14 moved; 17 scheduling conflict; 7 discharged; 4 withdrawn; 2 legal issues	Reimbursed for public transportation cost		Researchers provided the computers
	Chavez et al [44], 2020	30	One time	1-3 days	1 (3)		1 did not return	US $50		Researchers provided the headset
	Archie et al [47], 2018	55	Unclear	Unclear	Unclear		Unclear	Unclear		Unclear

^a^Percentage of dropouts: participants who were assessed at baseline and were lost to follow-up.

^b^N/A: not applicable.

^c^Unclear: authors did not provide sufficient or any information.

^d^ICT: information and communication technology.

^e^Linnemayr et al [38] and Tucker et al [39] reported the same population.

^f^The sample that was analyzed in Tucker et al [39] was included and analyzed in Linnemayr et al [38]. Dropout percentage was calculated from the 10 participants who were included in the intervention, the rest (18 participants) participated in focus groups and were not counted for dropout proportions.

### Technologies and Features

As illustrated in Table 2, a total of 8 (9 reports) [19,36,38-40,42,43,45,46] of the 11 studies provided e-mental health services, while the other 3 provided immersive guided meditation [44], access to health and vital resources [41], and educational content [47]. Regarding the type of ICTs, the most common were mobile apps designed for YEH (7 studies [19,36,40-43,46]). Other ICTs include video games for educational purposes [47], virtual reality for meditation [44], and computer exercises [45].

Almost all the studies provided technology to the participants. Only 1 study (2 reports) [38,39] required that participants own a phone that could receive SMS text messages. In this regard, the authors reported that phone and smartphone ownership was very elevated among the targeted population, finding that approximately 83.6% (183/219) of the youth already contacted had a phone that could receive SMS text messages and more than half (60/77, 78%) of their population owned a smartphone, and participants reported having phone plans with unlimited minutes and texts (54/77, 70%). A study that offered a virtual reality experience [44] reported that approximately 44% (8/18) of the participants under study had already used this technology in the past, and 100% (18/18) were interested in its future use.

Among the 8 studies that presented interventions to deliver e-mental health services through ICTs, 6 [19,36,40,42,43,46] combined e-mental health with mobile apps using the following features: trackers, push-in tips, automated systems to deliver support and brief cognitive behavioral interventions, or provided information about available resources for YEH. Five of these studies [19,36,40,42,46] also included sessions with a health provider or researcher (in person, phone or video calls, or text). One study (2 reports) in this category delivered e-mental health exclusively through SMS text messaging [38,39], and another study used computer exercises to deliver neurocognitive training [45].

### Feasibility

The feasibility of each intervention was assessed considering three main components [34]: (1) acceptability of the interventions among participants, described as the perceived usefulness of the intervention and the intention of use; (2) implementation characterized here as the frequency of use and the failures or issues presented during the intervention; and (3) efficacy considering the positive or negative outcomes reported by each study. The results are presented in Table 4.

In general, there was good acceptability of the use of ICT; the majority of studies (8/9, 89%) reported high or moderate to high ratings of participants’ evaluations regarding acceptability. Acceptability was rated according to the percentages of participants who reported high scores or gave good evaluations of the intervention (ie, high >80%, moderate 30%-70%, and low <30% of the participants).

Regarding implementation, 6 studies (7 reports) [19,38-40,42,43,46] provided data about the frequency of use; only 1 study [40] had moderate to low success, considering that a high number of participants dropped out (81%) of the intervention, while the rest reported moderate to high engagement. Frequency of use was rated considering the percentage of participants who completed >90% of the intervention (ie, high >90%, moderate 30%-80%, and low <30% of the participants). Most issues reported by the authors were technical problems with the technology, such as the need to replace phones, problems with data services, and phone charging. A study that used virtual reality [44] reported that some participants were concerned that the intervention may trigger seizures.

Some studies have also explored intervention efficacy, and the results are summarized in Table 4. The most frequent positive outcomes were related to improvements in emotion regulation and stress management (4 studies) [19,43-45]. This was followed by outcomes related to risky behaviors; 2 studies observed a decline in substance use [36,42] and risky sexual behavior [42], and 1 study [47] reported greater knowledge of marijuana use and its potential risks through the use of an educational video game. In addition, a study [45] found improvements in cognitive and psychological functioning after the use of computer exercises designed for neurocognition training, and another study [19] reported small changes in symptoms of depression and posttraumatic stress disorder.

Some studies [38,39,43] included participants’ self-reports regarding the benefits of the interventions. For instance, participants mentioned that push notifications helped them better regulate their emotions [43], and SMS text messages helped to overcome cravings [38,39].

**Table 4:** Feasibility of the interventions including acceptability, implementation, and intervention efficacy.

Intervention type, reference			Acceptability^a^	Implementation^b^		Efficacy^c^
				Frequency of use	Issues	
**Communication technologies**						
	Thompson et al [42], 2020	High		High	In all, 33% (1/3) participants discontinued the use of the app	The intervention group presented lower odds of drinking alcohol (OR^d^=0.14, 95% CI 0.03-0.64; *P*=.01) and having unprotected sex (OR=0.15, CI 0.03-0.85; *P*=.03) than the treatment as usual group, but no significant difference for use of marijuana (OR=0.39, CI 0.07-2.33; *P*=.30).
	Schueller et al [19], 2019	Moderate to high		Moderate to high	Mobile service provider, phone replacement, and exceeding the data limit	Very little improvements in clinical outcomes with small effect sizes for symptoms of depression (Cohen *d*=0.27), posttraumatic stress disorder (Cohen *d*=0.17), and emotion regulation (Cohen *d*=0.10); all *P*>.50.
	Glover et al [40], 2019	High		Moderate to low	23% (23/100) phone replacement	Unavailable
	Sheoran et al [41], 2016	High		Unavailable	Details unavailable	Unavailable
	Chao et al [36], 2017	Unavailable		Unavailable	Details unavailable	Intervention group showed significantly reduced use of marijuana (*P*<.05 ;from Chao et al [36]—poster)
	Karnik et al [46], 2017	High		High	Phone replacement	Unavailable
	Leonard et al [43], 2018	High		Moderate to high	Technical	Unavailable^e^
	Leonard et al [43], 2018	Moderate		Moderate	Phone charging and social issues (embarrassment)	Unavailable^e^
	Linnemayr et al^f^ [38], 2021	High		Moderate	Changed phone number, phone stolen, and bad reception	Unavailable
	Tucker et al^f^ [39], 2020	Moderate to high		High	Keeping the phone charged	Unavailable
**Other information and communication technologies**						
	Medalia et al [45], 2017	Unavailable		Unavailable	Sessions took longer than expected	Significant effect on cognitive (verbal memory: *F*_2,89_=20.28; *P*<.0001), processing speed (*F*_2,89_=9.35; *P*=.0002), executive functioning (*F*_2,89_=22.26; *P*<.0001), attention (*F*_2,89_=3.67; *P*=.03), global cognition (*F*_2,89_=39.89; *P*<.0001), and psychological functioning (*F*_2,89_=11.21; *P*<.00001)
	Chavez et al [44], 2020	High		Unavailable	Trigger seizures concern	No significant differences for anxiety or salivary cortisol measures between intervention (virtual reality meditation) and other groups (audio meditation and virtual reality imagery).
	Archie et al [47], 2018	High		Unavailable	Unavailable	Intervention group obtained significantly greater mean scores on knowledge about cannabis use and harms and psychosis (mean 6.8, SD 1.6 and 5.5, SD 1.9, respectively; *P*<.05)

^a^Acceptability as perceived usefulness of the intervention by youth and their practitioners, intention to use the technology—high: >80% of participants rated it with a high score or good evaluation; moderate: 30% to 70% of participants rated it with high scores or good evaluation; low: <30% of participants rated it with a high score or good evaluation.

^b^Implementation or feasibility framework: degree of success or failure of execution, amount and type of resources, and factors affecting implementation ease or difficulty. The feasibility study framework by Bowen et al [34]—high: >90% of allocated participants completed all or >90% of the sessions; moderate: 30% to 90% of allocated participants completed all or >90% of the sessions; <30% of allocated participants completed all or >90% of the sessions.

^c^Positive or negative outcomes tendency as reported by authors, statistically significant or not.

^d^OR: odds ratio.

^e^On the basis of qualitative methods the study reported that the participants perceived the app helpful in identifying and regulating emotions, and managing stress.

^f^Linnemayr et al [38] and Tucker et al [39] reported the same population.

### Quality Appraisal

Four randomized controlled studies [38,42,44,45] were included in the quality appraisal (Multimedia Appendix 2 [19,38-40,42-45]). Although the authors reported some results about the efficacy of the intervention, all of them were principally interested in the feasibility of the intervention. As a general overview, the main methodological issues were the lack of blindness and a lack of clarity on treatment allocation concealment and the follow-up of dropouts.

In addition, 4 nonrandomized experimental studies (quasi-experimental) were assessed for quality. However, only 2 [19,43] of them reported the effects of the interventions, while the other 2 [39,40] focused on feasibility. None of these studies provided a control group, and only 1 study provided information about the absence of coexisting treatments that may interfere with the cause-effect relationship. Finally, only 2 studies adequately described their follow-up, with no reported patterns of loss to follow-up (Multimedia Appendix 2).

## Discussion

### Principal Findings

This scoping review is the second in a series of reviews that we conducted at the intersections of health, technology, and YEH. We found a small number of studies conducted in even fewer cities within North America, indicating the limited research attention globally on the use of technology to deliver mental health services to YEH. This is rather concerning given that youth represent a significant part of the homeless population and that previous research indicates their high access to and use of technology for day-to-day needs, cumulatively indicating a largely unaddressed area of research and practice. Moreover, the geographical settings of the studies were unclear in some of the reviewed reports. For future research, we recommend more specific documentation regarding the location of the study to allow consideration of the role of contextual factors in relation to the results. Regarding the level of evidence, the studies included in this review were mostly pilot and feasibility studies, which were carried out to establish the preliminary planning of a full-size RCT and did not contribute to the assessment of the effectiveness and efficacy of the intervention [48]. According to the studies that provided sociodemographic information, the largest ethnicity was Black and African American; gender distribution was relatively balanced with a small representation of transgender and gender fluid individuals; and a small proportion of participants reported being homosexual, bisexual, or other. However, it is important to note that not all studies reported sufficient details regarding sample characteristics; for example, the mean age was not always reported, and only a few studies specified participants’ gender and sexuality. Moreover, the participants were not always characterized in terms of mental illness or condition.

The high levels of acceptability and success of implementation reported in these studies indicate that the use of ICTs to provide mental health services to YEH has the potential to be a feasible approach and warrants further research. Concurrently, although technology may support the delivery of mental health services, the use of a single app is unlikely to yield long-lasting results, especially considering that homelessness is a multifactorial problem. It is important to combine ICTs with traditional support and treatment services, which include the active participation of health care providers, as seen in certain studies [19,40,42,43,46]. This is consistent with previous literature, highlighting the need for ICTs to be implemented as cointerventions [26].

The most notable finding was that smartphones were the preferred type of ICT and were mostly used to offer services via mobile apps. Push notifications were particularly liked by YEH [19,40,43], suggesting that alerts and reminders in real time may be a promising way to engage and interact with YEH. Although a study concluded that SMS text messages also had high levels of acceptability [39], authors reported that during the first phase of the intervention, some participants did not appreciate the formality of the texts and described them as “mechanical” [39]. The authors then decided to make some changes to their SMS text messaging approach in the second phase of their research and found that participants preferred a light tone associated with the messaging; for example, with fun elements such as emojis and memes [38]. Daily trackers were another well-liked feature. The authors described this as helpful and beneficial for YEH [19,40,42,43]. This may be because of the ability of these tools to provide structure in daily life, which may contribute to a sense of empowerment and support. This aligns with the importance of providing self-management support to homeless people affected by chronic diseases [49]. Mobile technology increases access to multiple resources, which not only facilitates self-management but also responds to social needs [50]. Therefore, it is possible that smartphones and mobile apps, especially those that include reminders and daily trackers, have the potential to increase YEH outreach and engagement.

We also found emerging uses of 2 other types of technology in this population: virtual reality [44] and video games [47]. Despite the positive outcomes of these studies, the samples were small, and the interventions were in the early stages of development; thus, further research is needed to deepen our understanding of their feasibility and impact. Computer exercises for cognitive development were assessed in only 1 study [45], which indicated that the completion rate was particularly low, which may reflect how YEH’s challenging situation hinders their participation in programs that require their physical presence.

Overall, the findings from this review contribute to the broader literature situated at the intersections of homeless youth and technology access and use, which shows that youth use the internet, social media, and mobile technologies at high rates and for various social, informational, and daily needs [20,51]. Cumulatively, these findings indicate the importance of advancing the use of technology in research conducted with YEH as well as delivering mental health–related information and services to this population. More research is needed to better understand the use of technology to deliver mental health services to YEH as an adjunct to care for complementing face-to-face interventions or as stand-alone interventions and services.

Concurrently, it is important to note that this review also found several challenges encountered by the authors that pertain to technology that needs to be considered by practitioners and researchers embarking on this field. Issues pertain to access to wireless internet, battery life, and locations to charge batteries; hence, strategies to ensure technology availability and to guarantee access to the internet should be designed a priori. Moreover, limited attention was given to privacy and security, although confidentiality was a concern for some participants [41,43], and only 1 study [43] reported some information about observed adverse effects (embarrassment as a social issue). Future research should consider the potential risks and unfavorable effects of YEH [20]. Finally, according to our results and as suggested in previous systematic reviews [26], future research must consider looking beyond feasibility and start testing the effectiveness of ICT interventions in larger samples in terms of their ability to respond to the urgency of improving mental health care access and quality for YEH.

Finally, future research and practice in this area should consider including YEH more actively within the design, development, and evaluation of technology-enabled mental health interventions targeting YEH. This goes beyond including YEH as participants in usability studies and interviews or as employees to promote projects but more actively as research collaborators and partners (and the related challenges in this regard).

### Limitations of the Study

Despite the rigor of our methodology and the literature search, our results should be interpreted cautiously. First, this was a scoping review that only included 11 studies (12 reports), among which 3 were conference abstracts and posters, which provided limited information. Our results may be biased by the elevated percentages of dropouts [19,40,42,45] (43%, 81%, 33%, and 76%, respectively), and only a few studies have reported the reasons for dropout. Hence, it should be acknowledged that our results regarding acceptability and success of implementation levels may be overestimated if dropout percentages were mostly represented by participants who were dissatisfied with the interventions. The participants included in the RCT studies were not blinded to the interventions, which may have affected their perceptions, especially if they compared themselves with the control groups. All studies were published in the United States (Chicago, New York, Los Angeles, Oakland, and other nonspecified cities located in the northeast and midwestern states) and Canada, suggesting that our results may only be applicable to North America or in countries where the availability of high technology devices is more common and potentially also to urban areas with high rates of homelessness. In addition, few studies have reported the effectiveness of their intervention; therefore, we cannot provide any conclusions on this aspect.

### Conclusions

This scoping review synthesizes the current literature on the use of technology to provide mental health services to the YEH. These results are encouraging in terms of acceptability, suggesting that ICTs are promising tools to leverage in the delivery of mental health services to the YEH. We expect an increase in the use of ICTs in the coming years. Moreover, owing to the current COVID-19 pandemic and restrictive measures, an increase in mental health care needs among YEH may be observed, highlighting the importance of novel methods to reach and engage this population.

## Appendix

Multimedia Appendix 1 PRISMA-ScR (Preferred Reporting Items for Systematic Reviews and Meta-Analyses extension for Scoping Reviews) checklist.

Multimedia Appendix 2 Quality appraisal tables.

## Abbreviations

ICT: information and communication technology
PRISMA: Preferred Reporting Items for Systematic Reviews and Meta-Analyses
PRISMA-ScR: Preferred Reporting Items for Systematic Reviews and Meta-Analyses extension for Scoping Reviews
RCT: randomized controlled trial
YEH: youth experiencing homelessness

## Data Availability

Data sharing was not applicable to this paper, as all data were obtained from publicly available reports. The extracted data from these reports are presented in tables within the manuscript.
