# Supplementary material for: The Use of Technology to Provide Mental Health Services to Youth Experiencing Homelessness: Scoping Review
Source: J Med Internet Res. 2023 Jan 16;25:e41939. doi: 10.2196/41939 (PMC9887515; doi:10.2196/41939)
Supplement: Multimedia Appendix 2 [file jmir_v25i1e41939_app2.pdf]

Appendix 2. Quality appraisal tables.

Table S1. Quality appraisal non-randomized experimental studies according to the Joanna Briggs Institute critical appraisal checklist.

	Is it clear in the study what is the " cause" and what is the ' effect' (i.e. there is no confusion about which variable comes first)?	Were the participants included in any comparisons similar?	Were the participants included in any comparisons receiving similar treatment/care, other than the exposure or intervention of interest?	Was there a control group?	Were there multiple measurements of the outcome both pre and post the intervention/exposure?	Was follow up complete and if not, were differences between groups in terms of their follow up adequately described and analyzed?	Were the outcomes of participants included in any comparisons measured in the same way?	Were outcomes measured in a reliable way?	Was appropriate statistical analysis used?
Schueller et al., 2019	Yes	Yes	No	No	Yes	Unclear[a]	Yes	Yes	Yes
Glover et al., 2019	Yes	Yes	No	No	No	Yes	Yes	Yes	Yes
Leonard et al., 2018	Yes	Yes	Unclear[a]	No	No	No	N/A[b]	Yes	Yes
Tucker et al., 2020	Yes	N/A[b]	N/A[b]	No	No	Yes	Yes	Yes	N/A[b]

[a]Unclear: authors did not provide sufficient or any information.
[b]N/A: not applicable.

Table S2. Quality appraisal of randomized controlled trials according to the Joanna Briggs Institute critical appraisal checklist.

	Was true randomization used for assignment of participants to treatment groups?	Was allocation to treatment groups concealed?	Were treatment groups similar at the baseline?	Were participants blinded to treatment assignment?	Were those delivering treatment blinded to treatment assignment?	Were outcomes assessors blinded to treatment assignment?	Were treatment groups treated identically other than the intervention of interest?	Was follow-up complete and if not, were differences between groups in terms of their follow-up adequately described and analyzed?	Were participants analyzed in the groups to which they were randomized?	Were outcomes measured in the same way for treatment groups?	Were outcomes measured in a reliable way?	Was appropriate statistical analysis used?	Was the trial design appropriate, and any deviations from the standard RCT design (individual randomization, parallel groups) accounted for in the conduct and analysis of the trial?
Thompson et al., 2020	Yes	Unclear[a]	Yes	No	No	No	Yes	No	Yes	Yes	Yes	Yes	Yes
Chavez et al., 2020	Yes	Unclear[a]	No	No	No	No	Yes	N/A[b]	Yes	Yes	Yes	Yes	Yes
Medalia et al., 2017	Yes	Yes	Yes	No	No	No	Yes	Yes	Yes	Yes	Yes	Yes	Yes
Linnemayr et al., 2021	Yes	Yes	Unclear[a]	Unclear[a]	Unclear[a]	Unclear[a]	Unclear[a]	Unclear[a]	Yes	Yes	Yes	Yes	Yes

[a]Unclear: authors did not provide sufficient or any information.

[b]N/A: not applicable.
